# PCD 2021 Student Research Collection: Building Public Health Research Capacity in Real-World Settings and the 2022 Call for Papers

**DOI:** 10.5888/pcd18.210214

**Published:** 2021-07-08

**Authors:** Leonard Jack

**Affiliations:** 1 Preventing Chronic Disease, Office of Medicine and Science, National Center for Chronic Disease Prevention and Health Promotion, Centers for Disease Control and Prevention, Atlanta, Georgia

With this student collection, PCD celebrates the 10-year anniversary of our efforts to build scientific publishing skills and abilities among students. The primary aims of PCD’s student manuscripts have evolved over the years. Specifically, we aim to 1) provide an opportunity to become familiar with a journal’s manuscript submission requirements and peer review process; 2) foster connections among student knowledge and training, the conduct of quality research, and a journal’s publication expectations; 3) develop research and scientific writing skills to become producers of knowledge, rather than just consumers of knowledge; 4) provide an opportunity to become a first author on a peer-reviewed article; and 5) promote supportive, respectful, and mutually beneficial mentee relationships that strengthen students’ ability to generate and submit scholarly manuscripts throughout their professional careers (1). We believe that committing time, attention, and resources to providing student authors with valuable feedback (whether or not manuscripts are accepted) serves as a key capacity-building resource. Providing this feedback not only benefits PCD in the future but other peer-reviewed journals as well.

PCD published articles of winning student manuscripts from 2011 through 2015 and in 2017 and 2018. From 2011 through 2015, manuscripts were screened and reviewed by a panel of peer reviewers who identified an overall winner whose manuscript was ultimately published (2–11). Because of the tremendous response from students, we expanded submission screening, peer reviewing, and publishing to 5 student levels in 2017 and 2018, and winners were identified at the high school, undergraduate, graduate, doctoral, and postdoctoral levels (1,12). In addition to publishing student articles in each of the 5 levels, articles that successfully completed the peer-review process were also published. In this 2021 collection, we have continued to publish articles that successfully completed our rigorous peer-review process; however, we chose not to select a winner in each level. The COVID-19 pandemic rendered PCD unable to obtain the human resources necessary for facilitating a timely selection process. PCD will return to its usual selection process of identifying winners at all levels in future student competitions.

Over the years, PCD has refined eligibility requirements for students interested in submitting research manuscripts to the journal. Students must be a high school graduate, an undergraduate or graduate student, a medical resident, or a postdoctoral fellow. Authors must meet the journal’s criteria to be recognized as first author; that is, they must have prepared the first draft and conducted research on or practiced in the topic addressed in the manuscript. In addition, their work must have been completed within the previous 12 months. Manuscripts submitted for PCD consideration cannot be under consideration by another journal. PCD only considers Original Research or GIS Snapshots for student submissions. Most importantly, the author must serve in the combined roles of the manuscript’s first author and the corresponding author, which allows direct communication with the editor in chief and journal staff members. Students in this role receive critical instruction at every stage of scholarly publication, from submission, to peer review, to editorial revision, to production, and finally, to publication.

**Samuel F. Posner, PhD: Student Contest Visionary**

**Table:** 

The PCD 2021 Student Research Collection is dedicated to Samuel Posner, PhD, the person who envisioned and led the journal’s student competition in 2011. Dr Posner provided 9 years of service to PCD as editor in chief, from 2008 through 2016. Dr Posner’s legacy is one of scientific excellence, technical innovation, and service.	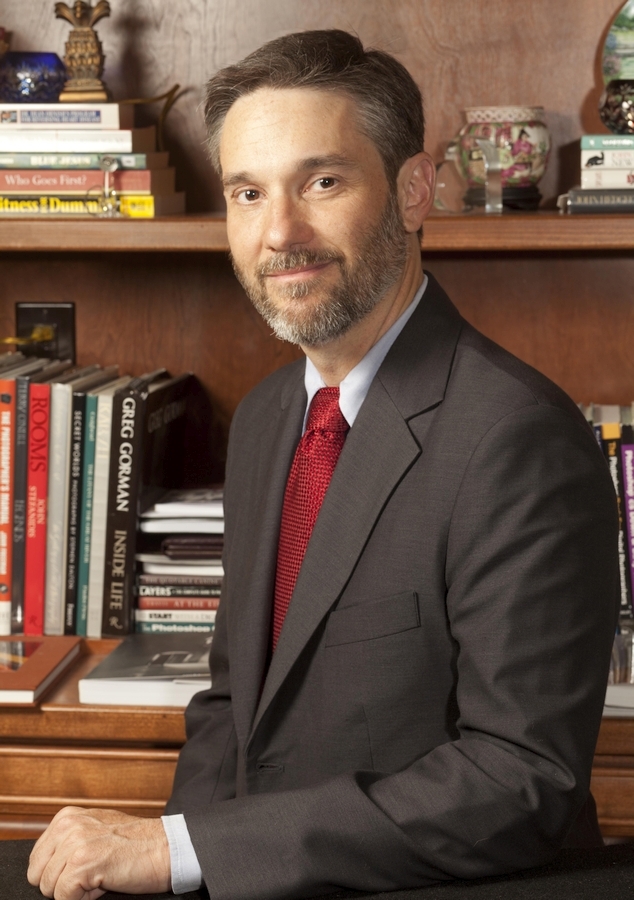
Dr Posner is now the Acting Director of the National Center for Immunization and Respiratory Diseases (NCIRD) and the center’s deputy director for science. In this role, he oversees the center’s surveillance, epidemiology, laboratory, and data science programs to expand and refine CDC’s capacity to detect, prevent, and respond to vaccine-preventable and respiratory infectious disease threats.	
Dr Posner came to CDC in 1998 from the University of California, San Francisco after completing doctoral work in quantitative psychology at the University of Southern California in 1996. He joined NCIRD in January of 2016 as the associate director for epidemiological science and director of the Office of Science and Integrated Programs. In this role, Dr Posner led the scientific review of several NCIRD programs, including the review and reframing of the Legionella program. Under his guidance, the Legionella program has grown in scope and reach and now funds 23 jurisdictions for both prevention and outbreak response.	
In the COVID-19 response, Dr Posner co-led the development of a scientific agenda to guide implementation of scientific activities for better understanding of COVID-19 transmission dynamics, risk factors for mortality, and prevention strategies. He also worked closely on the data and informatics architecture for the COVID-19 national vaccine program.	
Dr Posner is the author of more than 100 articles published in peer reviewed journals and has written more than 15 book chapters. He is an internationally recognized expert in the fields of preconception care and multiple chronic conditions, and he is an adjunct associate professor at both Emory University’s Rollins School of Public Health, Department of International Health, and the University of Alabama, Birmingham’s School of Public Health, Department of Health Behavior.	
Early in his career, Dr Posner recognized the value of mentoring students in the value of publication, and his student research competition brought a new generation of public health researchers and practitioners to PCD. Since the inception of the competition, the journal has received nearly 500 student manuscripts for consideration. Dr Posner’s vision to promote academic research excellence for students around the world lives on today.	

PCD’s student articles released to date address topics relevant to the prevention, screening, and surveillance of population-based interventions for chronic diseases, including but not limited to arthritis, cancer, diabetes, depression, obesity, and cardiovascular disease. Of the 38 manuscripts submitted for the PCD 2021 Student Research Collection, 10 successfully completed our rigorous internal and external peer-review process and multiple revisions. PCD sincerely appreciates our associate editors, the editorial board, members of the statistics review committee, and the many peer reviewers who provided detailed comments and suggestions to student authors. We congratulate each student author who developed and submitted a manuscript for consideration, whether it was accepted or not.

The current collection addresses a broad range of topics, including childhood obesity in secondary schools in Hong Kong (13); nutrition and physical activity among adults (14–16); the impact of inadequate sleep on mental health (17); the association between neighborhood built environments and depression in the rural South (18); colorectal cancer risk factors and screening among uninsured adults living in Tampa Bay, Florida (19); spatial accessibility to dental care among Alabama youth (20); identifying challenges to care for people living with hepatitis delta virus and their caretakers (21); and community resources to promote health among Chinese immigrants living in Philadelphia (22).

PCD is pleased to announce the Call for Student Papers: 2022 Publishing Opportunity for Students. Information about submission requirements are available on the PCD Announcements page at https://www.cdc.gov/pcd/announcements.htm. The deadline for submissions is Monday, March 28, 2022. PCD looks forward to continuing its commitment to the development of scientific writing and publishing skills among students. For more information about the journal and previous collections of student articles, please visit the PCD website at https://www.cdc.gov/pcd/index.htm.
